# Exosomal transfer of miR-769-5p promotes osteosarcoma proliferation and metastasis by targeting DUSP16

**DOI:** 10.1186/s12935-021-02257-4

**Published:** 2021-10-18

**Authors:** Wanshun Liu, Binyu Wang, Ao Duan, Kai Shen, Qi Zhang, Xiaolu Tang, Yongzhong Wei, Jian Tang, Sheng Zhang

**Affiliations:** 1 Department of Orthopedics, The First Affiliated Hospital of Nanjing Medical University, 300 Guangzhou Road, Nanjing, 210029 Jiangsu China; 2 Department of Pain Management, Sir Run Run Hospital, Nanjing Medical University, Nanjing, 211100 China; 3 Department of Hematology, The First Affiliated Hospital of Nanjing Medical University, 300 Guangzhou Road, Nanjing, 210029 Jiangsu China; 4 Department of Plastic and Burn Surgery, The First Affiliated Hospital of Nanjing Medical University, 300 Guangzhou Road, Nanjing, 210029 Jiangsu China; 5 Nanjing Shuangjian Medical Technology Co., Ltd, Nanjing, 210029 Jiangsu China

**Keywords:** BMSCs, Osteosarcoma, Exosomal, miR-769-5p, DUSP16, EMT

## Abstract

**Background:**

Osteosarcoma (OS) is a malignant tumor originating from mesenchymal stem cells, and has an extremely high fatality rate and ability to metastasize. Although mounting evidence suggests that miR-769-5p is strongly associated with the malignant progression and poor prognosis of various tumors, the exact role of miR-769-5p in OS is still unclear. Therefore, this study aimed to explore the relationship between miR-769-5p and the malignant progression of OS, and its underlying mechanism of action.

**Methods:**

miR-769-5p expression was analyzed in GSE28423 from the GEO database and measured in OS clinical specimens and cell lines. The effects of miR-769-5p on OS proliferation, migration and invasion were measured both in vivo and in vitro. In addition, bioinformatics analyses and luciferase reporter assays were used to explore the target genes of miR-769-5p. Rescue experiments were also conducted. Moreover, a co-culture model was used to test the cell interaction between bone mesenchymal stem cells (BMSC) and OS cells.

**Results:**

We found that miR-769-5p is highly expressed in OS clinical specimens and cell lines. In vivo and in vitro experiments also showed that miR-769-5p significantly promoted the proliferation, migration and invasion of OS cells. Dual-specific phosphatase 16 (DUSP16) was negatively associated with miR-769-5p expression in OS cells and tissue samples and was validated as the downstream target by luciferase reporter assay and western blotting. Rescue experiments showed that DUSP16 reverses the effect of miR-769-5p on OS cells by negatively regulating the JNK/p38 MAPK signaling pathway. Additionally, the results of the co-culture of BMSCs and OS cells confirmed that miR-769-5p was transferred from BMSCs to OS cells through exosomes.

**Conclusions:**

In summary, this study demonstrates for the first time that BMSC-derived exosomal miR-769-5p promotes OS proliferation and metastasis by targeting DUSP16 and activating the JNK/p38 MAPK signaling pathway, which could provide rationale for a new therapeutic strategy for OS.

**Supplementary Information:**

The online version contains supplementary material available at 10.1186/s12935-021-02257-4.

## Introduction

Osteosarcoma (OS) is a high-grade malignant tumor originating from mesenchymal cells, which takes up about 2% of all pediatric tumors and 20% of bone neoplasms [1]. Although improved surgical techniques and neoadjuvant chemotherapy are available, the 5-year overall survival of OS is just 60–70% [2]. About 20% of OS patients already have lung metastases when they are diagnosed, with the 5-year overall survival rate of < 30% [3]. Consequently, elucidating the OS metastatic mechanism is of great importance.

MicroRNAs (miRNAs), the RNA molecules containing 22–28 nucleotides with no protein-encoding ability, can bind to 3′-untranslated region (UTR) in target mRNA, thus promoting mRNA degradation and inhibiting its translation [4]. miRNAs have important functions in tumor initiation and malignant transformation [5]. Moreover, miRNAs regulate many pathological processes in OS, like proliferation, apoptosis and epithelial-mesenchymal transition (EMT) [6]. miR-769-5p up-regulation is reported in many tumors [7, 8]. Nevertheless, miR-769-5p’s effect on OS remains incompletely clear.

EMT is a dynamic process where polarized epithelial cells acquire a mesenchymal phenotype [9]. This promotes tumor cell metastasis to distant sites during the development of malignant tumors [10, 11]. Moreover, EMT is involved in the malignant progression of OS [12].

Initially identified as the JNK/p38 protein inhibitor, Dual Specificity Phosphatase 16 (DUSP16) binds to JNK scaffold protein and negatively regulates JNK activation [13, 14]. DUSP16 loss is correlated with tumor progression [15], but its effect on OS development and the association of miR-769-5p/DUSP16 axis with JNK/p38 MAPK signaling in OS development remain unclear.

Bone mesenchymal stem cells (BMSCs) are bone marrow progenitors with multipotential differentiation and self-renewal abilities [16]. BMSCs have been indicated to participate in tumor progression. However, their effect on tumor growth is controversial [17–19]. BMSCs promote the development of breast cancer and prostate cancer [17], yet inhibit the progression of glioblastoma and hepatocellular carcinoma [18, 19]. Moreover, OS cells may originate from BMSCs, while BMSCs regulate OS metastasis [20]. However, the mechanism by which BMSCs regulate the malignant progression of OS remains unclear. Exosomes are important pathways for intercellular communication, they are extracellular vesicles with the diameter of 30–150 nm, which can carry proteins, lipids and RNA [21]. Moreover, growing evidence shows that BMSC-derived exosomes can mediate OS progression by transferring non-coding RNAs [22, 23]. However, the relationship between BMSCs, exosomes and OS has not been fully investigated.

In this work, we explored the role of miR-769-5p in the malignant progression of OS through in vivo and in vitro experiments, and revealed the underlying mechanism. We found that miR-769-5p transferred from BMSCs through exosomes can promote OS proliferation and metastasis by down-regulating DUSP16 and activating the JNK/p38 MAPK pathway. Our research reveals a new potential mechanism for the progression of OS and provides a new strategy for the treatment of OS.

## Materials and methods

### Clinical specimens

Altogether 64 OS patients undergoing tumor biopsies before chemotherapy and radiotherapy at Department of Orthopedics, First Affiliated Hospital of Nanjing Medical University during 2015–2021 were enrolled in this study. Intraoperative tumor specimens were histologically confirmed by three pathologists, and tumor and adjacent normal samples were stored in liquid nitrogen. Informed consent was provided by each patient. Our study was approved by The Evaluation Committee and Ethics Committee of First Affiliated Hospital of Nanjing Medical University. (Additional file 1: Table S1) shows patient clinicopathological information.

### Cells and cell culture

Human osteoblasts (hFOB1.19) and human OS cells (HOS, MG63, 143B, Saos-2, U2OS) were provided by Cell Bank of Type Culture Collection of the Chinese Academy of Sciences (CBTCCCAS, Shanghai, China). OS cells were cultured in DMEM (Gibco, CA, USA) containing 10% fetal bovine serum (FBS, Gibco, NY) and 1% penicillin/streptomycin (P/S) (Gibco) under 37 °C, whereas hFOB1.19 cells were cultivated within DMEM containing 10% FBS and 1% P/S under 33.5 °C. BMSCs purchased from CBTCCCAS were cultured in DMEM (Gibco) containing 10% FBS (Gibco, NY) and 1% P/S (Gibco) at 37 °C. Afterwards, all cells were incubated under 5% CO2 condition.

### Lentivirus construction establishment and transfection

Lentiviral vectors (GenePharma, Shanghai, China) were utilized to construct LV2-hsa-miR-769-5p mimic/inhibitor vectors (miR-769-5p mimics/inhibitor). Negative controls with LV2 empty lentivirus (miR-769-5p mimics NC, miR-769-5p inhibitor NC) were also constructed. OS cells reaching 70–80% confluency were infected with lentiviral vectors (miR mimics, miR inhibitor, and corresponding NCs). Cells were treated with puromycin for 1 week to obtain stable cell lines. miR-769-5p vector transfection efficiency was verified by qRT-PCR. Vectors with DUSP16 over-expression and down-regulation containing puromycin-resistant sequence was constructed by GenePharma (Shanghai, China) through lentiviral gene transfer, with scrambled lentiviral construct as negative reference. OS cells were transfected with lentiviral vectors (DUSP16, vector control, sh-DUSP16 and sh-NC). DUSP16 expression was confirmed by qRT-PCR and Western blotting (WB).

### Cell co-culture

To observe cell interactions between BMSCs and OS cells, OS cells were seeded into 6-well plates (corning, USA), whereas BMSCs were seeded into the upper chamber of a 0.4-μm co-culture chamber (Millipore, USA). The co-culture chambers were then inserted into the plate wells. After 48 h of co-culture, OS cells at the plate bottom were trypsinized, then proliferation, migration and invasion were measured.

### Exosome extraction and identification

BMSCs were cultured to passage 3–4, and medium was replaced by DMEM containing 10% exosome-free FBS to culture for another 72 h. Supernatants were harvested and cells were removed by centrifugation (300 × g, 10 min, 4 °C; then, 2000 × g, 10 min, 4 °C) to remove cell debris. Afterwards, supernatant were obtained for centrifugation (10,000 × g, 30 min, 4 °C) to removing great membrane vesicles. Later, the resultant supernatants were placed into ultracentrifuge tube for centrifugation (120,000 × g, 70 min, 4 °C). After removing supernatants, the bottom pellet was resuspended in PBS, followed by centrifugation (140,000 × g, 90 min, 4 °C). The bottom pellet was resuspended in PBS to obtain exosomal samples. Exosome morphology was identified by transmission electron microscopy (TEM). Exosome size distribution was analyzed by nanoparticle tracking, whereas exosomal protein markers were detected by WB.

### Exosome uptake by MG63 and 143B cells

Dil dye (Molecular Probes, USA) was incubated with exosomes at room temperature to label exosomes. Excess dye was removed by centrifugation (100,000 × g, 60 min, 4 °C), followed by PBS rinsing thrice. Dil-labeled exosomes were resuspended in DMEM medium containing 10% exosome-free FBS, and co-cultured with OS cells for 24 h. Cells were washed thrice with PBS, fixed with 4% paraformaldehyde for 15 min, and permeabilized with 0.1% Triton X-100 for 5 min. The nuclei were stained with DAPI solution. Exosome internalization by MG63 and 143B cells was measured under the laser confocal microscope.

### RNA extraction and qRT-PCR

TRIzol reagent (Invitrogen, Carlsbad, California, USA) was adopted for extracting total cellular and tissue RNAs. RNA concentration and quality were measured by NanoDrop spectrophotometer (ND-100, Thermo Fisher Scientific). miRNA and exosomal RNA were extracted using RNeasy/miRNeasy Mini Kit (Qiagen) and exosomal RNA and protein extraction kit (Thermo Fisher Scientific, USA). qRT-PCR was then performed as described previously [24]. mRNA and miRNA expression levels were normalized to β-actin and U6, respectively. Every assay was conducted thrice. Relative gene expression was determined by 2^ − ΔΔCT approach. (Additional file 2: Table S2) lists primer sequences.

### Western blotting (WB)

The cells were lysed with lysis buffer for 10 min on ice, then the BCA protein quantification kit (Thermo Fisher Scientific, USA) were used to determine the protein concentration. Exosomal RNA and protein extraction kits (Thermo Fisher Scientific, USA) were used to extract exosomal proteins. Electrophoresis is performed after the protein is boiled and denatured. After blocking with fast blocking solution for 30 min, the PVDF membrane were incubated with the primary antibody overnight at 4 °C, and then incubated with the secondary antibody for 1 h at room temperature. ECL reagent (Millipore, USA) were utilized to exposure the membrane using a Tanon 4200 automatic chemiluminescence imaging analysis system. Primary antibodies utilized are shown in (Additional file 3: Table S3).

### Colony formation assay

The cells were digested to make a suspension and counted; 1,000 cells were seeded in each well of a 6-well plate. The medium was changed every 3 days. After 10 days, the cells were fixed with 4% paraformaldehyde for 15 min, then stained with crystal violet solution for 30 min and photographed with a digital camera, and the clone formation rate was calculated.

### CCK-8 assay

The cells were digested to make a single cell suspension and counted to 2 × 10^4^/ml, 100 μl cells are spread in 96-well plates peer well. Each sample was set up with 6 multiple holes, and 100 μl PBS was added to the edge holes. 10 μl of CCK8 (Dojindo,

Japan) was added to each well at day 1 to 5, after incubation for 2 h, the OD value of 450 nm was detected by the microplate reader.

### 5-Ethynyl-2-deoxyuridine (EdU) incorporation assays

100,000 cells were seeded to each well in 12-well plates and incubated for 24 h until they were fully confluenced, then the medium was removed and 1 ml 1 × EdU reagent (RiboBio, China) were added to each well and incubated for 2 h. Subsequently, 1 ml of 4% paraformaldehyde was added to each well and fixed at room temperature for 15 min. 1 ml 0.5% TritonX-100 was added to each well for 10 min and 400 μl 1 × Apollo staining reaction solution was incubated with cells for 30 min at room temperature. Finally, cell slides were taken out and stained the nucleus with oily DAPI and mount the slide. An upright fluorescence microscope (Olympus, Japan) is used for taking pictures.

### Scratch assay

For the scratch assay, OS cells were cultured in a 6-well plate, when the density reached 80%, a 200 μl tip was used to gently scribble vertically to ensure that there were no cells at the scribe. The scribed cells were washed away with PBS, and then serum-free medium was added to the plates. Scratch pictures were taken under a phase-contrast microscope (Olympus, Japan) at 0 h and 24 h, and the migration of cells in each group were analyzed with Image J software.

### Transwell assays

For transwell migration assay, Cells were trypsinized and counted. A total of 2 × 10^4^ cells was cultured in the upper chamber of the Transwell chamber (8 μm pore size; Costar, NY, USA) with 200 μl of serum-free medium, 700 μl medium with 10% FBS was added to the lower chamber of the culture plate. Cells were cultured at 37 °C, 5% CO2 for 24 h. The chamber was fixed with 500 μl 4% paraformaldehyde at room temperature for 20 min, and then stained with 500 μl crystal violet solution for 30 min. Cells in the up-chamber were wiped off and the chamber was washed with deionized water, followed by taken pictures with a microscope (Olympus, Japan). For the invasion assay, transwell chamber pre-coated with Matrigel was used to detect the cell invasion ability, and the experimental method was consistent with the Transwell migration experiment.

### Luciferase reporter assay

Binding sites between DUSP16 and miR-769-5p were predicted by TargetScan database (http://www.targetscan.org/vert_72/). GenScript (Nanjing, China) synthesized wild-type DUSP16 (WT-DUSP16-3 UTR) and mutant DUSP16 (MUT-DUSP16-3 UTR). MG63 and 143B cells were transfected with miR-NC or miR-769-5p mimic and later co-transfected with WT-DUSP16-3’ UTR and MUT-DUSP16-3’ UTR for 48 h. Luciferase activities were measured by the dual-luciferase detection kit (Solarbio, China).

### Immunohistochemistry (IHC)

Clinical samples and nude mouse tumor samples were subjected to 4% paraformaldehyde fixation, paraffin embedding, and slicing to 4-µm sections. After antigen retrieval and blocking, tissue sections were incubated with anti-DUSP16 and anti-Ki-67 primary antibodies overnight at 4 °C, and then with secondary antibodies under ambient temperature for 1 h. Fresh 3,3-diaminobenzidine solution was applied in the sections. Five random fields were selected for measuring positive tumor proportion and staining intensity.

### Animal experiments

Altogether 20 six-week-old female BALB/c athymic nude mice were randomly divided into four groups (MG63-miR mimics, 143B-miR inhibitor, and corresponding NCs; n = 5 per group) for subcutaneous tumor formation experiments. Nude mice were injected with luciferase-expressing stably transfected OS cells in 200 μl OS cell suspension (2 × 10^7^/ml) via the front right armpit. Tumor volume was determined every four days by volume = (width)^2^ × length/2. The IVIS imaging system (Caliper Life Sciences, USA) was used to detect tumor fluorescence intensity on day 28 after cell implant. At the end of the experiment (day 28), the nude mice were euthanized by injecting 150 mg/kg sodium pentobarbital into the tail vein, and the tumors were dissected for weighing and IHC. In addition, 20 six-week-old female BALB/c athymic nude mice were used to generate OS lung metastasis models by the above grouping strategy. Nude mice were injected with luciferase-expressing stably transfected OS cells (2 × 10^7^/ml, 100 µl) via the tail vein. On day 28 after cell implant, lung metastasis fluorescence intensity was detected by IVIS imaging system. After euthanasia on the 28th day, lung tissues were resected from nude mice, sliced into sections and stained with HE.

### Statistical analysis

Data were displayed as mean ± SD. Each experiment was repeated independently thrice. Statistical analysis was completed by SPSS22.0 (SPSS Inc., Chicago, Illinois, USA). Comparisons between two groups were analyzed by student’s t-test, while those among multiple groups were examined by one-way ANOVA. P < 0.05 indicated statistical significance.

## Results

### miR-769-5p is highly expressed in OS tissues and cells

**Fig. 1:**
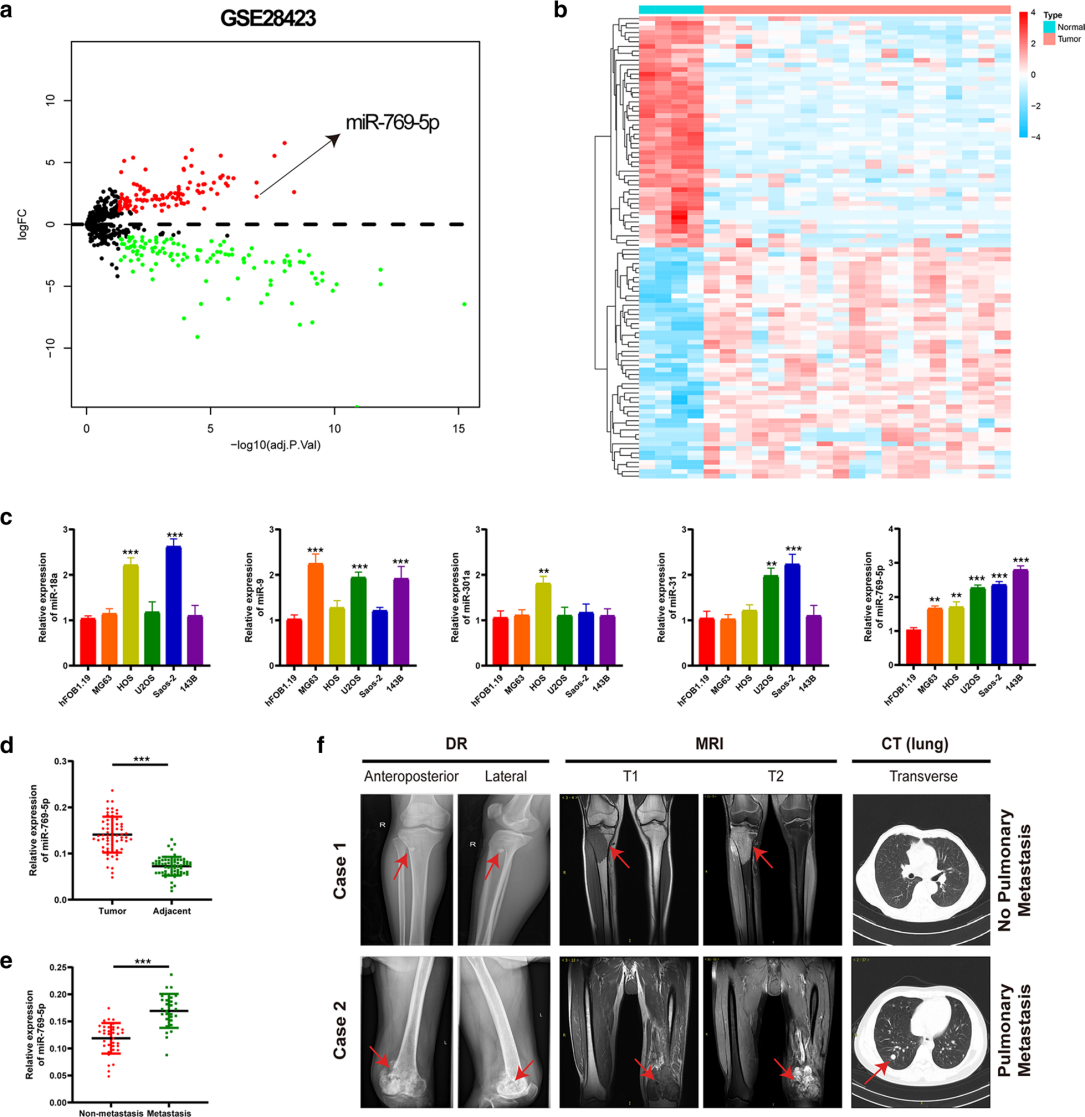
miR-769-5p is highly expressed in OS tissues and cells. **A** Volcano plot compared the differentially expressed miRNAs between OS cell lines and Human osteoblast cell line (hFOB1.19) from GSE28423; **B** The cluster heat map showed the up-regulated and down-regulated microRNAs (miRNAs) in GSE28423; **C** qRT-PCR detected miR-18a, miR-9, miR-301a, miR-31, miR-769-5p expressions in OS cell lines and hFOB1.19; **D** qRT-PCR detected miR-769-5p expression in 64 pairs of clinical OS specimens and matched adjacent normal tissues; **E** The expression level of miR-769-5p in patients with and without pulmonary metastasis; **F**Representative X-ray, computed tomography (CT) and Magnetic resonance imaging (MRI) images of OS patients with and without pulmonary metastasis. Data are presented as the means ± SD. *P < 0.05, **P < 0.01, ***P < 0.001

R package limma [25] was utilized to analyze OS dataset (GSE28423). A volcano map (Fig. 1A) and a heat map (Fig. 1B) of significant differentially expressed miRNAs were generated (The logFC > 1 and adj. P. Value < 0.05 indicated statistical significance). (Additional file 4: Table S4) lists the top five up-regulated miRNAs in OS cells. The corresponding expression was measured within 5 distinct OS cell lines and hFOB1.19 cells (Fig. 1C). As a result, miR-769-5p expression markedly increased within OS cells. We therefore chose miR-769-5p for further research. miR-769-5p levels were measured in 64 pairs of tumor and adjacent samples by qRT-PCR. The results showed that the expression level of miR-769-5p in tumor samples was significantly higher than adjacent normal tissues (Fig. 1D). Moreover, miR-769-5p expression in lung metastasis patients significantly higher than non-lung metastasis patients (Fig. 1E). Representative images of patients with and without lung metastases are shown in Fig. 1F. Furthermore, the relationship between clinicopathological characteristics of OS patients and miR-769-5p expression was analyzed. miR-769-5p expression was significantly positively correlated with tumor size, tumor-node-metastasis stage, and lung metastases (Additional file 1: Table S1).

### miR-769-5p promotes the proliferation of OS cells in vitro and in vivo

**Fig. 2:**
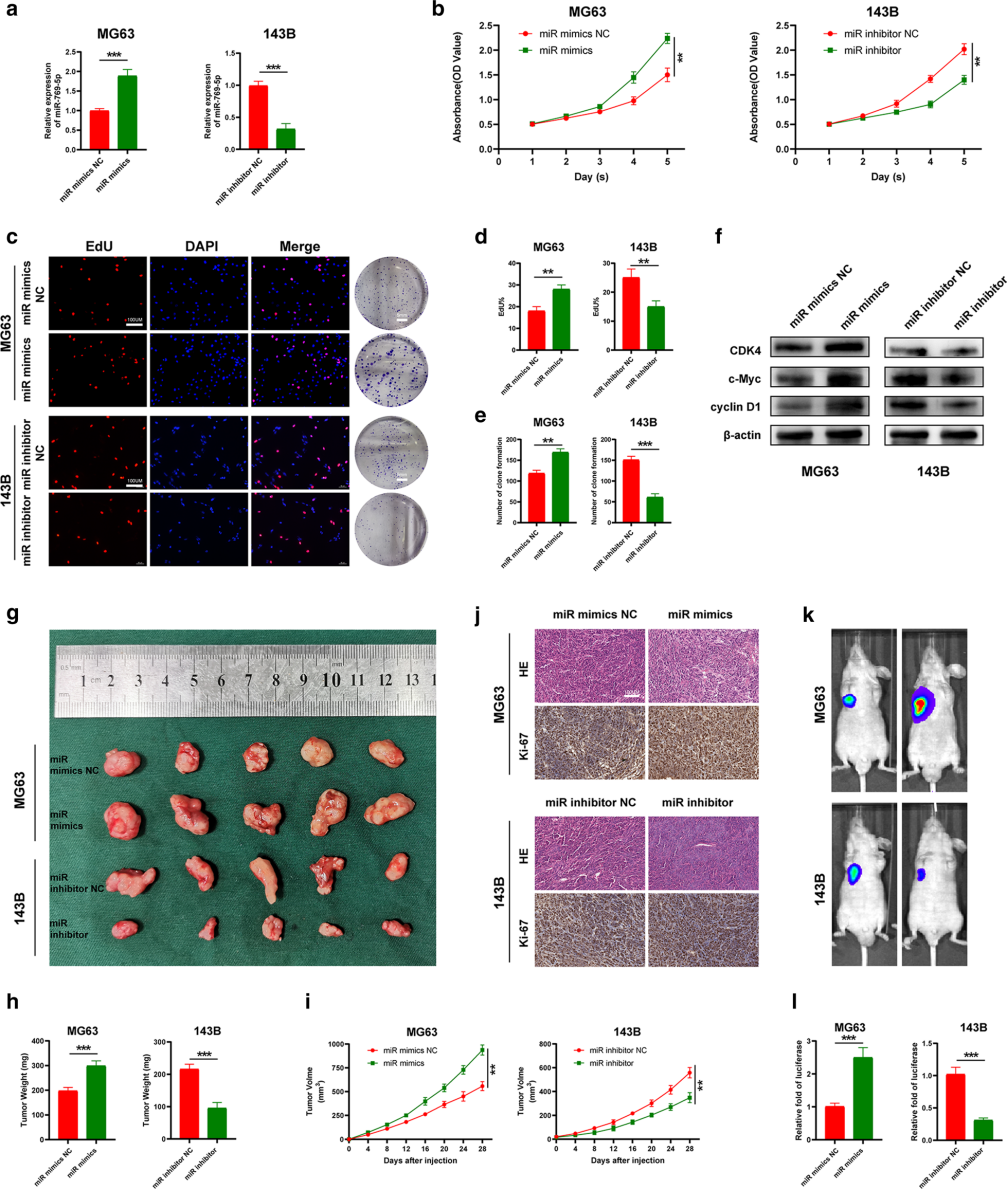
miR-769-5p promotes the proliferation of OS cells in vitro and in vivo. **A** The expression of miR-769-5p in MG63 and 143B cells transfected with miR-769-5p mimics or miR-769-5p inhibitor; **B-E** CCK-8 (**B**), EdU (**C**, **D**) and colony-formation assays(**C**, **E**) were used to detect the effect of miR-769-5p on proliferation in vitro; **F** Western blot analysis of cell-cycle-related proteins following miR-769-5p mimics and inhibitor; **G** Images of tumors obtained from mice treated with miR-769-5p mimics and miR-769-5p inhibitor and their negative controls; n = 5 mice/group; **H**, **I** Tumor weight (**H**) and volume (**I**) were calculated; n = 5 mice/group; **J** Representative H&E-stained tumors and immunohistochemical staining of Ki-67 from mice in different groups; n = 5 mice/group; **K** Representative images of tumors were obtained by the IVIS imaging system; n = 5 mice/group; **L** Quantification of the luciferase is shown. Data are presented as the means ± SD. *P < 0.05, **P < 0.01, ***P < 0.001

To study the role of miR-769-5p in OS proliferation, we transfected MG63 cells with miR-769-5p mimic and the 143B cell line with the miR-769-5p inhibitor (Fig. 2A). The results of CCK8 assay showed that cell proliferation was significantly promoted in the miR-769-5p mimics group while inhibited in the miR-769-5p inhibitor group (Fig. 2B). Moreover, EdU assay indicated that miR-769-5p plays a key role in cell proliferation, as the percentage of mitotic cells was increased in the miR-769-5p mimics group and vice versa (Fig. 2C, D). Furthermore, the clonality of OS cells was increased in the miR-769-5p mimics group but suppressed in the miR-769-5p inhibitor group (Fig. 2C, E). Based on WB results, miR-769-5p mimics remarkably promoted G1/S checkpoint protein levels, whereas miR-769-5p inhibitor had opposite effect (Fig. 2F). For better studying miR-769-5p’s function in OS proliferation in vivo, transfected MG63 and 143B cells were subcutaneously injected into nude mice. Clearly, miR-769-5p mimic group had markedly increased tumor weight and volume relative to mimic NC group, while those were lower in miR-769-5p inhibitor group than inhibitor NC group (Fig. 2G–I). HE and IHC staining on tumor specimens suggested that Ki-67 expression was augmented in miR-769-5p mimic group, whereas opposite result was observed in miR-769-5p inhibitor group (Fig. 2J). Moreover, in vivo imaging experiments showed consistent results (Fig. 2K, L).

### miR-769-5p promotes the metastasis of OS cells in vitro and in vivo

**Fig. 3:**
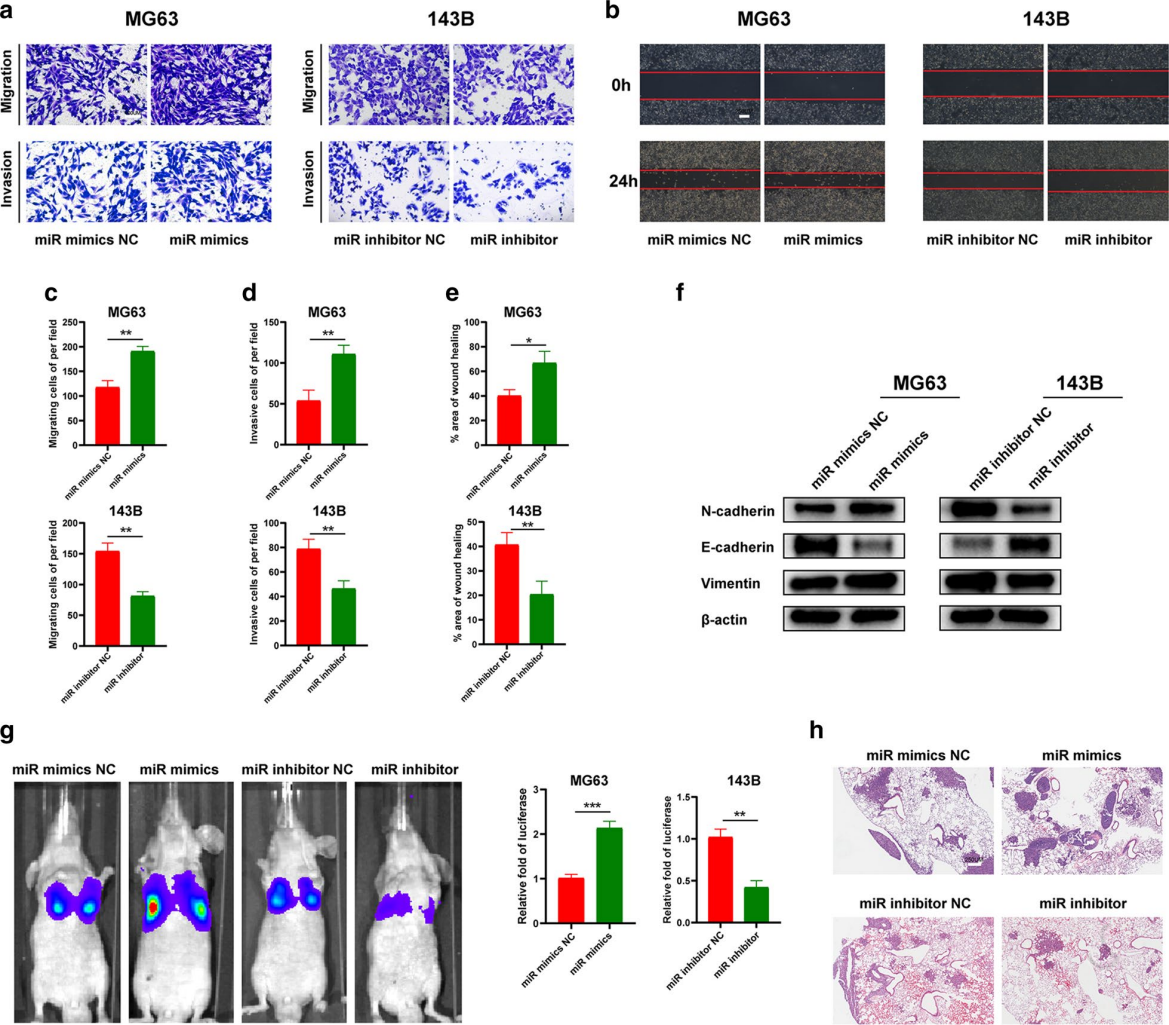
miR-769-5p promotes the metastasis of OS cells in vitro and in vivo. **A-E** Transwell migration (**A**, **C**), Transwell invasion (**A**, **D**) and Scratch assays (**B**, **E**) were used to evaluate the effect of miR-769-5p on cell migration and invasion; **F** Western blot analysis of Epithelial-mesenchymal transition (EMT)-related proteins following miR-769-5p mimics and inhibitor; **G** Representative images of pulmonary metastases were obtained by the IVIS imaging system. Quantification of the luciferase is shown; n = 5 mice/group; **H** Representative H&E-stained lung sections from mice in different groups; n = 5 mice/group; Data are presented as the means ± SD. *P < 0.05, **P < 0.01, ***P < 0.001

To explore the role of miR-769-5p in OS metastasis, Transwell migration and invasion assays were conducted. miR-769-5p mimics markedly increased the number of migrated cells and enhanced the invasive ability of cells, whereas miR-769-5p inhibitor had opposite effect (Fig. 3A, C and D). Scratch assay showed that the migration rate was increased in the miR-769-5p mimics group while decreased in the miR-769-5p inhibitor group (Fig. 3B, E). According to WB analysis, miR-769-5p mimics significantly increased N-cadherin and vimentin expression and decreased E-cadherin expression. Conversely, miR-769-5p inhibitor decreased N-cadherin and vimentin expression, and increased E-cadherin expression (Fig. 3F), indicating that miR-769-5p promoted OS metastasis by activating EMT. An OS lung metastasis model was used in vivo to clarify miR-769-5p’s function. A nude mouse lung metastasis model was constructed and mice were divided into four subgroups (miR mimics, miR inhibitor, and corresponding NCs). After six weeks, miR-769-5p mimic group had significantly more lung metastases, while miR-769-5p inhibitor group had fewer lung metastases, as revealed by in vivo imaging experiments (Fig. 3G). HE staining of nude mouse lung metastases confirmed the observed metastases (Fig. 3H). Collectively, in vitro and in vivo data supported that miR-769-5p promoted OS metastasis by activating EMT.

### DUSP16 is down-regulated in OS and is a downstream target gene of miR-769-5p

**Fig. 4:**
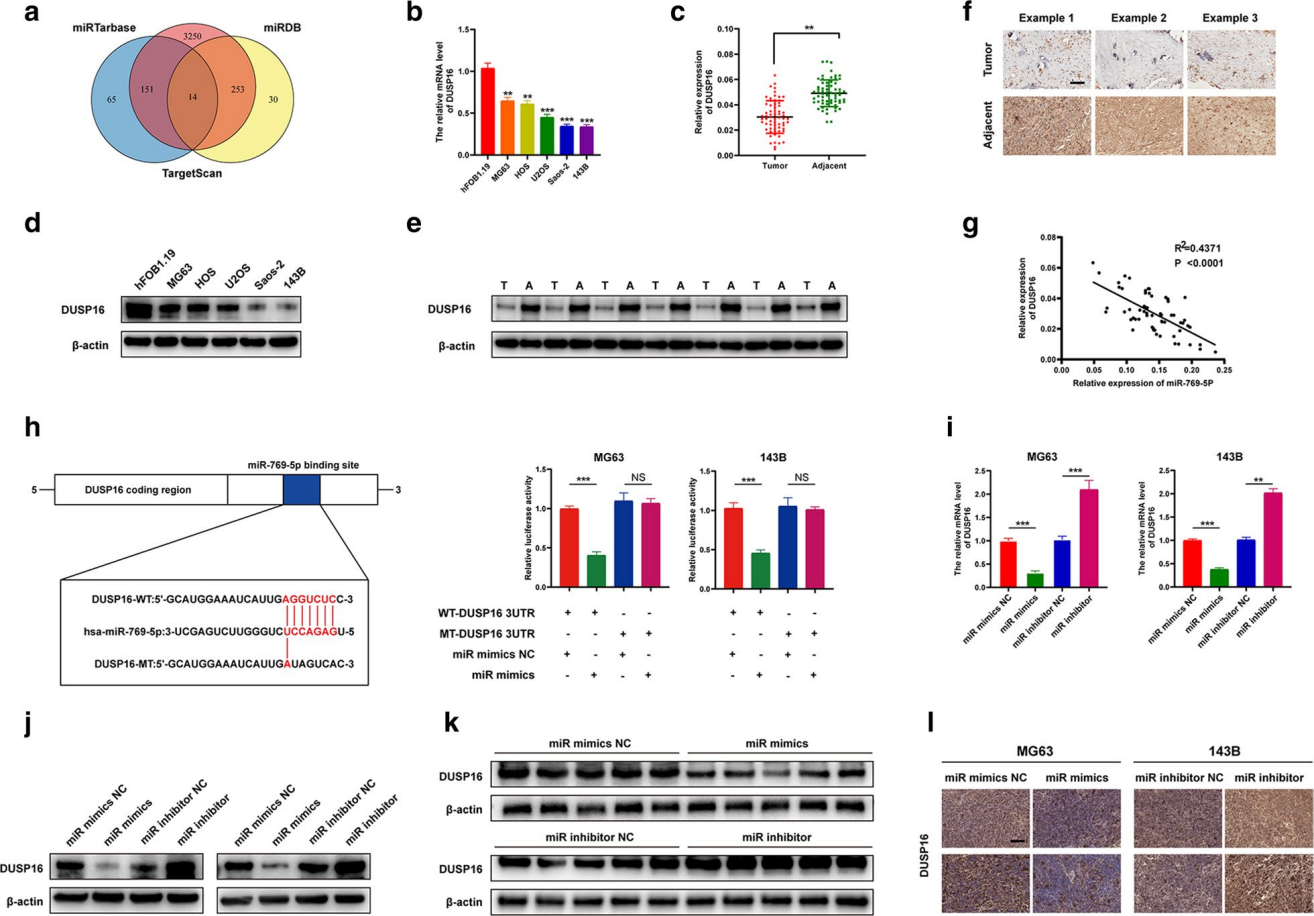
DUSP16 is down-regulated in OS and is a downstream target gene of miR-769-5p. **A** Bioinformatics analysis showed that hsa-miR-769-5p has a total of 14 target genes in miRDB, miRTarBase, and TargetScan; **B, C** The mRNA expression level of DUSP16 in OS cell lines and hFOB1.19 (**B**) and clinical samples (**C**); **D**, **E** The protein level of DUSP16 in OS cell lines and hFOB1.19 (**D**) and clinical samples (**E**); **F** Representative immunohistochemical staining of DUSP16 between OS tissues and adjacent tissues; **G** Negative correlation between miR-769-5p and DUSP16 expression in OS tissues; **H** Luciferase reporter assay was performed to confirm that miR-769-5p directly bound to the 3′-UTR region of DUSP16. Luciferase activity was analyzed in OS cells co-transfected with miR-769-5p mimics or negative control with pGL3-DUSP16-WT or pGL3- DUSP16-MUT; **I**, **J** The expression of DUSP16 in OS cells after alteration of miR-769-5p expression was detected by qRT-PCR (**I**) and western blot (**J**); **K**, **L** Western blot (**K**) and immunohistochemical analysis (**L**) were performed to evaluate DUSP16 expression in vivo; n = 5 mice/group. Data are presented as the means ± SD. *P < 0.05, **P < 0.01, ***P < 0.001

To explore the specific mechanism of the role of miR-769-5p in metastasis, the miRTarBase, miRDB and TargetScan databases were analyzed to predict downstream targets (Fig. 4A). Among Fourteen candidate target genes, DUSP16 was chosen for further study because it was involved in various tumors and plays a role in inhibiting tumor progression [26]. qRT-PCR and WB assays were performed on 6 cell samples and 64 pairs of clinical samples. DUSP16 expression markedly decreased within OS tissues and cells (Fig. 4B–E). DUSP16 IHC in tumor tissues further corroborated this conclusion (Fig. 4F). Moreover, DUSP16 was negatively correlated with miR-769-5p in OS clinical samples (Fig. 4G). As revealed by (Additional file 1: Table S1), DUSP16 was negatively correlated with clinicopathological factors like tumor size, tumor-node-metastasis staging, and lung metastasis. TargetScanHuman 7.2 (http://www.targetscan.org/vert_72/) were used to predict targeted relationship of miR-769-5p with DUSP16 [27]. Based on dual luciferase reporter assay, miR-769-5p over-expression significantly reduced the wild-type DUSP16 luciferase activity, but had little effect on mutant DUSP16 (Fig. 4H). DUSP16 mRNA and protein expression was down-regulated with miR mimics and up-regulated by miR inhibitors (Fig. 4I, J). In addition, IHC and WB analyses on nude mouse tumor specimens supported the above experimental conclusions (Fig. 4K, L), indicating that DUSP16 was a downstream target of miR-769-5p with down-regulation in OS.

### miR-769-5p promotes the metastasis and proliferation of OS cells by targeting DUSP16

**Fig. 5:**
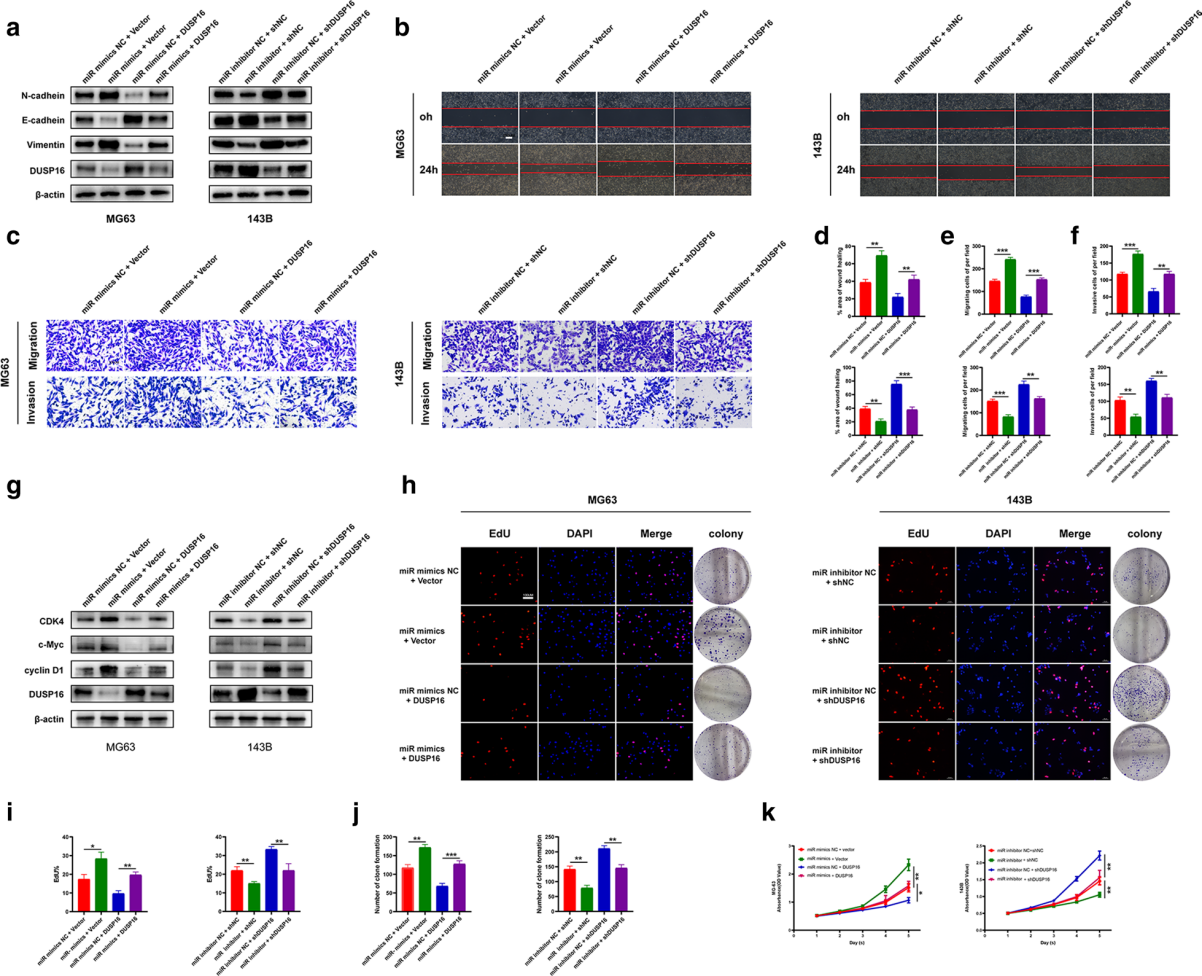
miR-769-5p promotes EMT and proliferation by suppressing DUSP16 in OS cells. **A** Western blot analysis was conducted to evaluate the EMT-related protein levels. Rescue experiments for miR-769-5p mimics were conducted via the ectopic expression of DUSP16 in MG63 cells. Rescue experiments for miR-769-5p inhibitor were conducted by downregulating DUSP16 in 143B cells; **B-F** Rescue experiments were conducted using the Scratch assay (**B**, **D**), transwell migration assay (**C**, **E**) and transwell invasion assay (**C**, **F**); **G** Western blot analysis was also conducted to evaluate the cell-cycle-related proteins level; **H–K** Rescue experiments were also conducted using the EdU assay (**H**, **I**), clone formation assay (**H**, **J**) and CCK-8 assay (**K**). Data are presented as the means ± SD. *P < 0.05, **P < 0.01, ***P < 0.001

Rescue experiments were conducted to confirm whether miR-769-5p promoted the malignant progression of OS by targeting DUSP16. According to WB results, DUSP16 overexpression decreased vimentin and N-cadherin expression and increased E-cadherin expression (Fig. 5A). Moreover, DUSP16 overexpression restored the effect of miR-769-5p mimics. Based on Scratch assay, DUSP16 overexpression reduced the positive effect of miR-769-5p mimics on OS cell migration and sh-DUSP16 rescued the effects of miR-769-5p inhibitor on cell migration (Fig. 5B, D). According to Transwell assays, DUSP16 inhibited OS cell migration and invasion and rescued the impact of miR-769-5p on them (Fig. 5C, E and F). Furthermore, DUSP16 overexpression significantly reduced CDK4, c-Myc and cyclin D1 expression, and significantly restored the impact of miR-769-5p mimics on OS proliferation (Fig. 5G). DUSP16 significantly inhibited OS proliferation and rescued the impact of miR-769-5p mimics in EdU (Fig. 5H, I), colony formation (Fig. 5H, J) and CCK8 (Fig. 5K) assays, while sh-DUSP16 reduced that of miR-769-5p inhibitor. Collectively, miR-769-5p promoted OS cell proliferation and EMT by specifically down-regulating DUSP16, which was reversed with DUSP16 overexpression.

### miR-769-5p regulates the JNK/p38 MAPK signaling pathway by targeting DUSP16

**Fig. 6:**
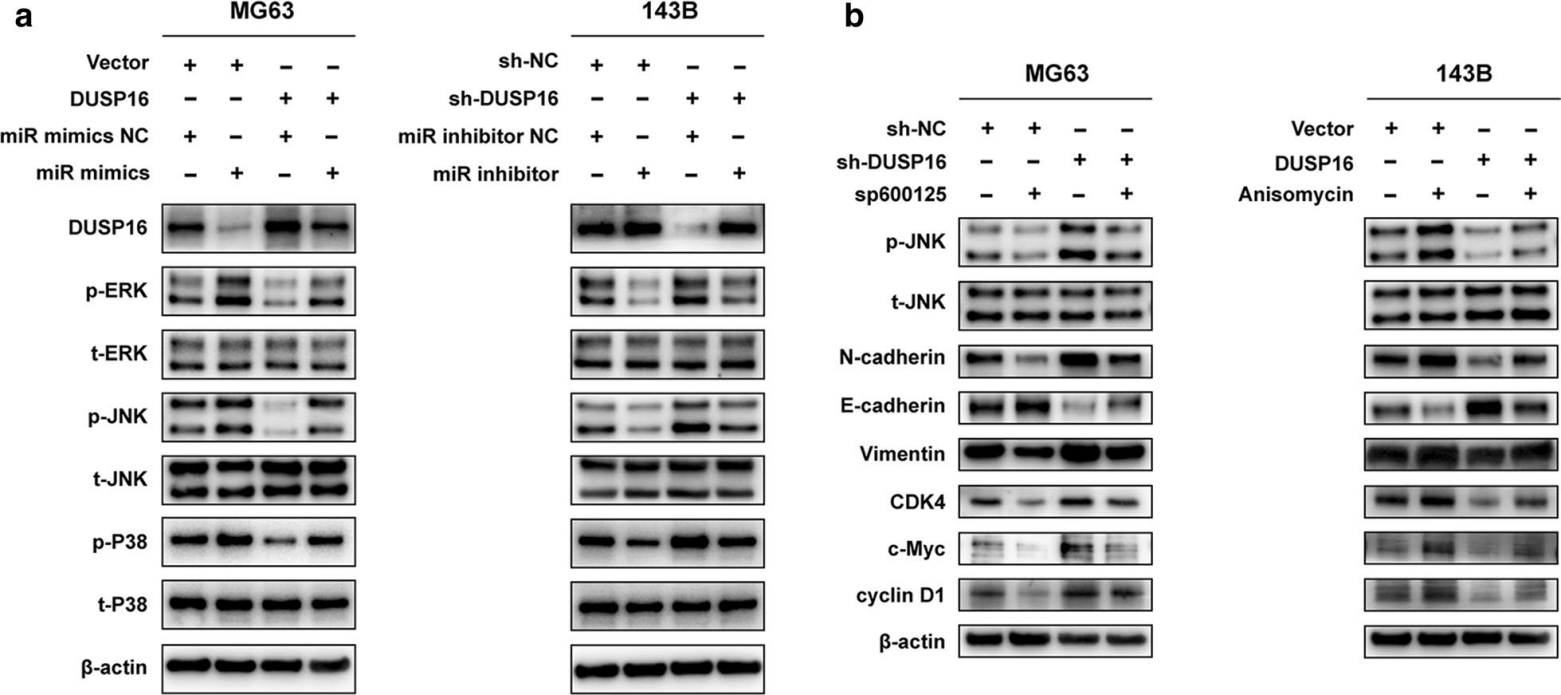
miR-769-5p regulates the JNK/p38 MAPK signaling pathway by targeting DUSP16. **A** Representative images of western blot analysis of p-JNK, JNK, p-ERK, ERK, p-P38, P38 and DUSP16 levels in transfected MG63 and 143B cells; **B** Representative images of western blot analysis of p-JNK, JNK, N-cadherin, Vimentin, E-cadherin, c-Myc, CDK4 and cyclin D1 levels in transfected MG63 and 143B cells treated with the JNK inhibitor sp600125 and the JNK activator Anisomycin, respectively

The JNK/p38 MAPK pathway is closely related to the invasion and metastasis of a variety of tumors. Numerous published studies have shown that DUSP16 can inhibit the MAPK signaling pathway [28]. Therefore, the relationship between miR-769-5p/DUSP16 axis and OS metastasis and proliferation via JNK/p38 MAPK axis is of great interest. miR-769-5p mimics significantly increased the expression of p-p38, p-ERK and p-JNK proteins expression, while DUSP16 diminished this effect. miR-769-5p inhibitors significantly reduced p-p38, p-JNK and p-ERK levels, while sh-DUSP16 rescued these effects (Fig. 6A). To determine whether miR-769-5p induced EMT and OS proliferation through JNK/p38 MAPK signaling pathway, OS cells were treated with JNK agonists (Anisomycin) and inhibitors (SP600125). WB suggested that sh-DUSP16 significantly increased EMT-related proteins and G1/S checkpoint proteins expression, and SP600125 reversed these effects. Anisomycin rescued the inhibition of DUSP16 on EMT and OS cell proliferation (Fig. 6B), supporting that miR-769-5p regulated the JNK/p38 MAPK signaling pathway by targeting DUSP16 and promoted OS progression.

### Exosomes released by BMSC promote EMT and the proliferation of OS cells

**Fig. 7:**
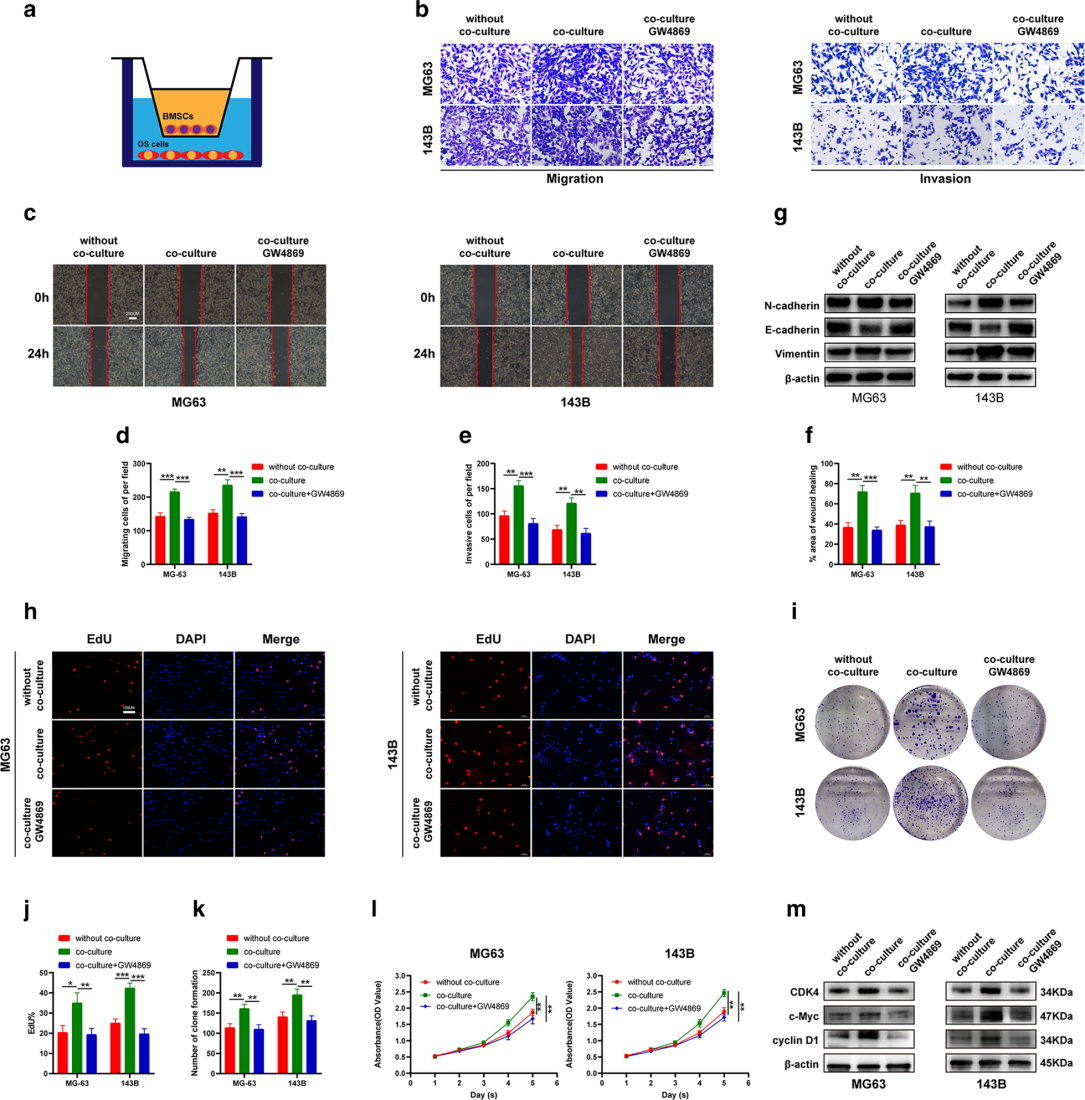
Exosomes released by BMSC promote EMT and the proliferation of OS cells. **A** Diagrammatic sketch of BMSCs and OS cell coculture; **B-F** The effect of BMSCs with or without treatment of GW4869 on OS cell migration and invasion was evaluated by Transwell migration (**B**, **D**), Transwell invasion (**B**, **E**) and Scratch assays (**C**, **F**); **E** Western blot analysis of EMT-related proteins following cocultured with BMSCs with or without treatment of GW4869; **H-I** The effect of BMSCs with or without treatment of GW4869 on OS cell proliferation was evaluated by EdU (**H**, **J**), colony-formation (**I**, **K**) and CCK-8 assays(**L**). **M** Western blot analysis of cell-cycle-related proteins following cocultured with BMSCs with or without treatment of GW4869. Data are presented as the means ± SD. *P < 0.05, **P < 0.01, ***P < 0.001

BMSCs, the OS precursor cells, participate in OS progression through cell-to-cell interactions [20]. BMSC exosomes play important roles in this process, but the specific mechanism remains unclear. To further explore the specific mechanism, BMSCs and OS cells were cultivated in the upper and lower chambers, respectively. The chamber diagram is shown in Fig. 7A. According to Transwell migration (Fig. 7B, D), Transwell invasion (Fig. 7B, E) and Scratch assays (Fig. 7C, F), OS cells had markedly increased migration and invasion ability after co-culture, but GW4869 (exosome secretion inhibitor) rescued the effect. Through WB, co-culturing OS cells with BMSCs significantly up-regulated vimentin and N-cadherin expression, but down-regulated E-cadherin expression, whereas GW4869 counteracted such effect (Fig. 7G). The effect of co-culturing OS cells with BMSCs on proliferation was also analyzed, which revealed that co-culture improved EdU incorporation (Fig. 7H, J), colony formation (Fig. 7I, K) and positive results in CCK-8 assay (Fig. 7L), but was repressed with GW4869. Through Western blotting showed that co-culturing OS cells with BMSCs significantly increased G1/S checkpoint protein expression, which was counteracted with GW4869 (Fig. 7M).

### BMSC-derived exosomes can be taken up by OS cells

**Fig. 8:**
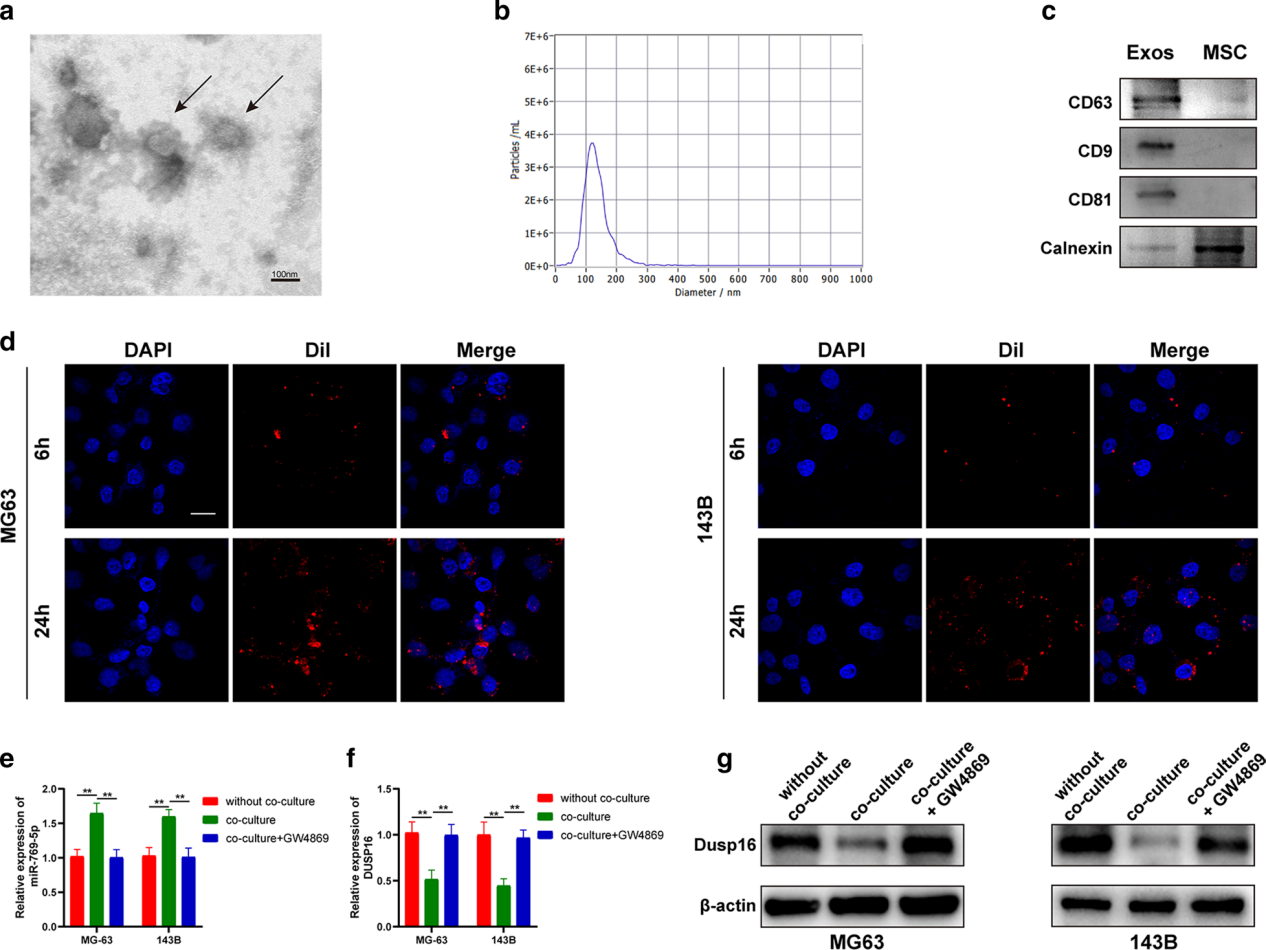
BMSC-derived exosomes can be taken up by OS cells. **A** Transmission electron micrograph of exosomes derived from BSMCs; **B** Nanoparticle tracking analysis of the diameter and concentration of exosomes; **C** Exosomal biomarkers protein were further examined by western blot; **D** Uptake of Dil-labelled exosomes by MG63 and 143B cells was detected; **E**, **F** The mRNA expression level of miR-769-5p (**E**) and DUSP16 (**F**) was detected by qRT-PCR in different groups; **G** The expression of DUSP16 protein was detected by western blotting in different groups. Data are presented as the means ± SD. *P < 0.05, **P < 0.01, ***P < 0.001

TEM, Nanoparticle Tracking and WB analyses were used to identify BMSC-derived exosome morphology, diameter and protein composition (Fig. 8A-C). Dil-labeled BMSC-derived exosomes were taken up by MG63 and 143B cells under confocal microscope (Fig. 8D). Through qRT-PCR, miR-769-5p expression in co-cultured group significantly increased, which was inhibited by exosome release inhibitors (Fig. 8E). Moreover, based on WB and qRT-PCR, DUSP16 expression decreased after co-culture (Fig. 8F, G). Therefore, we hypothesize that exosomal miR-769-5p transferring from BMSCs were taken up by OS cells to down-regulate DUSP16.

### miR-769-5p derived from BMSCs exosomes promotes the metastasis and proliferation of OS cells

**Fig. 9:**
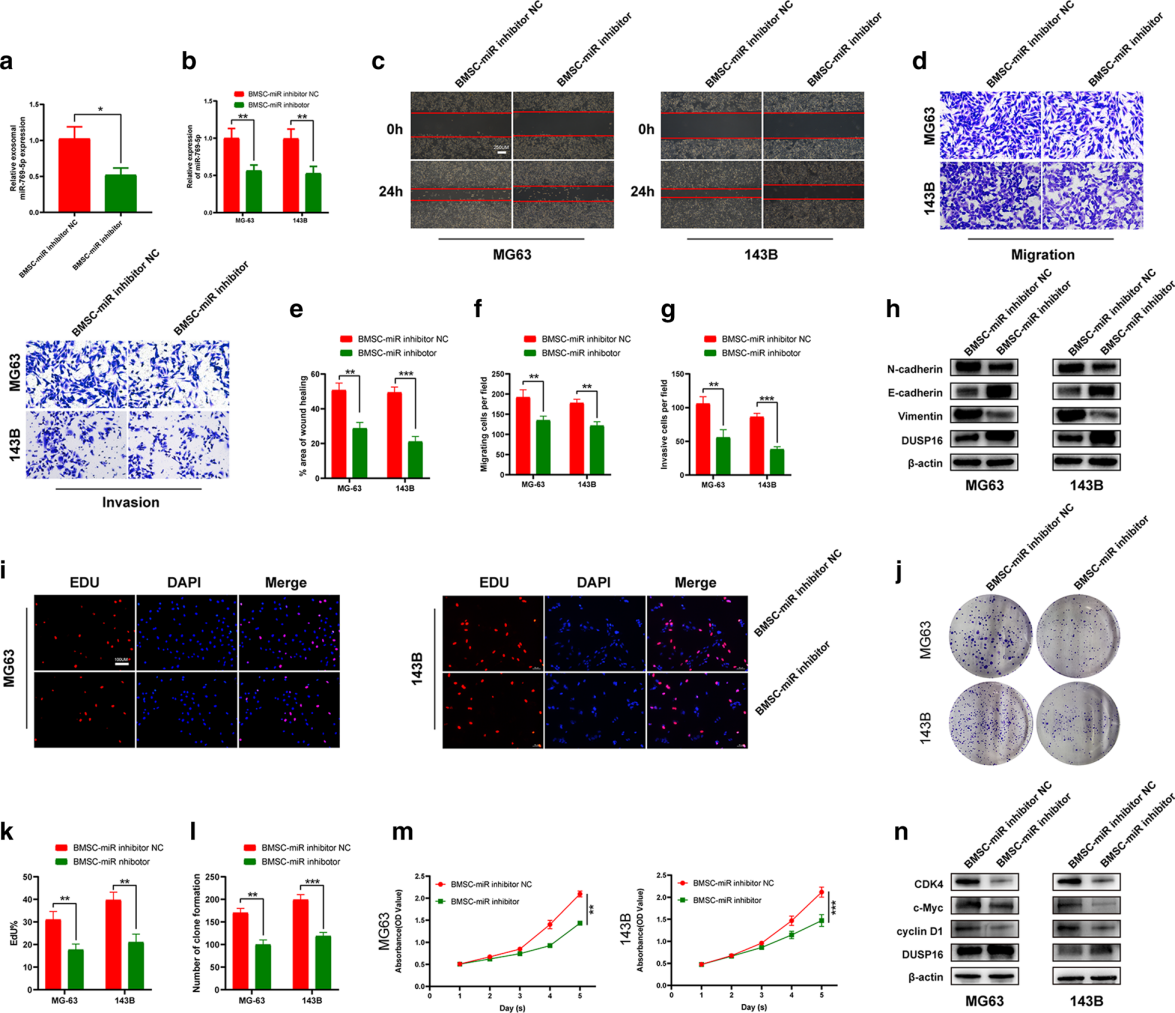
miR-769-5p derived from BMSCs exosomes promotes the metastasis and proliferation of OS cells. **A** The expression of miR-769-5p in BMSC-derived exosomes with or without transfected miR-769-5p inhibitor; **B** Relative miR-769-5p expression of OS cells cocultured with miR-769-5p inhibitor-BMSCs compared to BMSCs were detected by qRT-PCR; **C-G** cocultured OS cells migration and invasion abilities was evaluated by Scratch (**C**, **E**), Transwell migration (**D**, **F**) and Transwell invasion assays (**D, G**); **H** EMT-related proteins were detected by western blotting. **I-M** cocultured OS cells proliferation ability was evaluated by EdU (**I**, **K**), colony-formation (**J**, **L**) and CCK-8 assays (**M**); **N** Cell-cycle-related proteins were detected by western blotting. Data are presented as the means ± SD. *P < 0.05, **P < 0.01, ***P < 0.001

To confirm our hypothesis, BMSCs were treated with miR-769-5p inhibitors. By qRT-PCR, miR-769-5p levels within BMSC exosomes decreased (Fig. 9A). miR-769-5p levels within BMSCs co-cultured OS cells were also detected, it was found that miR-769-5p level significantly decreased in miR-769-5p inhibitor group (Fig. 9B). miR-769-5p inhibitor-treated BMSCs decreased OS cell migration and invasion in co-culture system, as revealed by Scratch (Fig. 9C, E), Transwell migration (Fig. 9D, F) and Transwell invasion assays (Fig. 9D, G). Moreover, EMT-related protein expression reduced after miR-769-5p inhibitor treatment (Fig. 9H). In addition, miR-769-5p inhibitor-treated BMSCs decreased OS proliferation in co-culture system, as revealed by EdU (Fig. 9I, K), colony-formation (Fig. 9J, L) and CCK8 assays (Fig. 9M). G1/S checkpoint-related proteins were also inhibited (Fig. 9N).

**Fig. 10:**
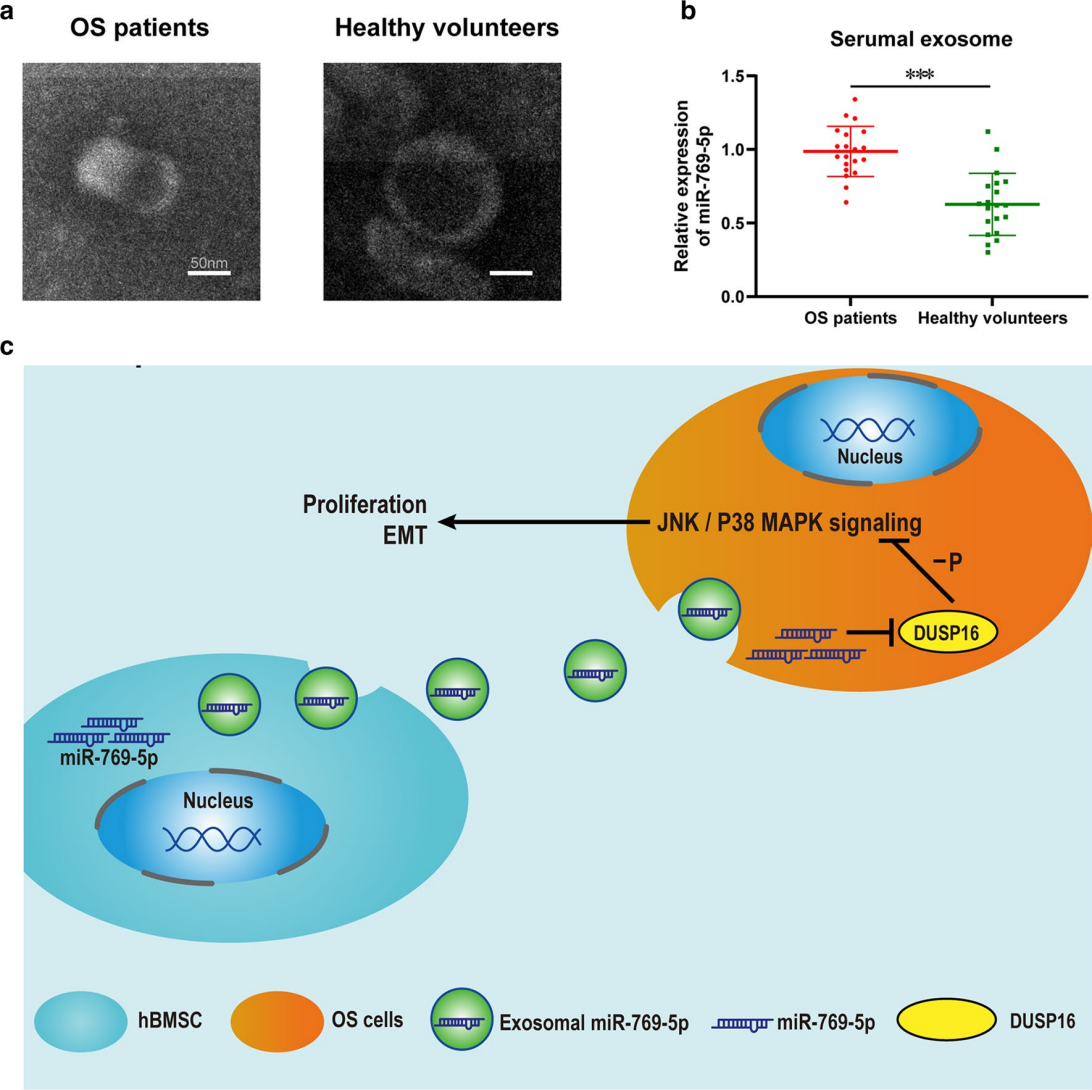
Identification of Exosomes in the Serum of Patients and Graphical Abstract. **A** Transmission electron micrographs of exosomes derived from OS patients and healthy volunteers; **B** The expression of miR-769-5p in serum exosomal of OS patients compared to volunteers; **C** miR-769-5p derived from BMSCs exosomes promotes OS cells migration, invasion and proliferation by downregulating DUSP16 and then activating the JNK/P38 MAPK signaling pathway. Data are presented as the means ± SD. *P < 0.05, **P < 0.01, ***P < 0.001

To verify exosomal miR-769-5p’s role as the OS clinical marker, serum exosomes were extracted from OS patients and healthy volunteers (Fig. 10A), it was discovered that OS cases had markedly higher serum exosomal miR-769-5p expression than normal volunteers (Fig. 10B). In summary, BMSCs promoted OS cell progression by releasing exosomes containing miR-769-5p (Fig. 10C). The flowchart is shown in (Additional file 5: Fig S1).

## Discussion

OS, a highly fatal and metastatic bone tumor [29], brings great distress to patients and their families. However, treatments have not significantly improved recently and the prognosis remains dismal [30]. Therefore, it is crucial to explore the pathogenesis and formulate new treatments [31, 32]. BMSCs participate in tumor genesis and progression [33]. Moreover, exosomes have vital functions in intracellular information transmission, which are intricately linked to cell migration, differentiation and invasion [34]. miRNAs occupy 70% of exosomal non-coding RNAs and play an important role in diverse tumor occurrence and development [35]. Therefore, the role of exosomes in OS requires further analysis. This study found that miR-769-5p transferring from BMSC-derived exosomes promoted OS progression by targeting DUSP16 and activating the JNK/p38 MAPK signaling.

Since the discovery, miRNAs are closely related to many processes like tumor proliferation, apoptosis and metastasis [36]. Recently, miRNAs are anti-tumor therapeutic targets and potential diagnostic biomarkers [37]. Moreover, miRNAs have also been implicated in the malignant progression of OS [38]. miR-769-5p is previously found to be up-regulated in various tumors and predicts poor prognosis [39, 40]. For example, miR-769-5p is up-regulated in gastric cancer, promotes cancer cell proliferation, and inhibits apoptosis [39]. Human skin fibroblast exosomes-derived miR-769-5p exacerbates ultraviolet radiation-induced bystander effect through cell-to-cell communication [41], signifying that miR-769-5p participates in intracellular interactions. It was discovered that miR-769-5p expression was up-regulated in OS cells through bioinformatics analysis and its expression in OS cells and tissues was verified. Based on our in vitro and in vivo data, miR-769-5p significantly promoted OS cell proliferation and EMT. Clinical data from OS patients indicated the relationship of miR-769-5p up-regulation with dismal prognosis.

EMT represents a key process where tumor cells obtain migration and invasion capacities, and it relates to distant metastases [42, 43]. Studies have demonstrated that miRNAs regulate the ability to form distant metastases by various tumor types by influencing the EMT process [44]. miR-769-5p increased OS cell invasion and migration by regulating EMT, and DUSP16 was a candidate target gene for miR-769-5p.

DUSP16, also known as MAPK phosphatase 7 (MKP7), is a specific dephosphorylated protein of threonine/tyrosine residues in MAPK protein family [28]. DUSP16 is down-regulated in different tumors and predicts disease prognosis [26]. Therefore, DUSP16 expression was measured in OS cells and tissues and it was found that DUSP16 expression markedly decreased. Dual-luciferase reporter assays and rescue experiments confirmed that DUSP16 functioned as the miR-769-5p downstream target gene in OS. However, the downstream miR-769-5p/DUSP16 axis should be further explored.

The JNK/p38 MAPK signaling pathway plays a vital role in tumor genesis and progression [45]. DUSP16 inhibits JNK-mediated signaling events by dephosphorylating threonine and tyrosine residues in the JNK activation ring and effectively prevents downstream effector activation [46]. Therefore, whether miR-769-5p activates JNK/p38 MAPK signaling pathway by targeting DUSP16 should be explored. WB indicated that miR-769-5p up-regulation promoted p-ERK, p-JNK and p-p38 expression, which was rescued by DUSP16. DUSP16 down-regulation significantly restored the effect of miR-769-5p inhibitor on the JNK/p38 MAPK signaling pathway. The relationship between this pathway and OS progression was analyzed. JNK activator rescued the inhibition of DUSP16 on EMT-related proteins and proliferation-related proteins, indicating that DUSP16 regulated EMT and OS proliferation by regulating this pathway.

BMSCs, as the precursor cells of OS, play a crucial role in tumor progression [47]. Moreover, exosomes participate in intercellular communication during pathophysiological progression through transmitting genetic material (such as miRNAs) to other cells [48]. Furthermore, BMSCs-derived exosomal miR-208a enhances OS cell proliferation and invasion [49], while BMSCs-derived exosomal miR-206 inhibits OS progression by targeting TRA2B [50]. There are a great number of similar studies, which further demonstrate that exosomal miRNAs shuttled from BMSCs is possibly related to OS development. For confirming BMSCs exosomes’s function in OS, we co-cultured BMSCs with OS cells and found that BMSC exosomes play a key role in OS progression. Moreover, after down-regulating miR-769-5p in BMSCs, the tumor-promoting effect of BMSC exosomes on OS was restored, which suggested that BMSCs played a vital role in promoting the malignant progression of OS through exosomal miR-769-5p.

## Conclusion

In summary, our results first suggest that the BMSC exosomes-derived miR-769-5p promotes OS proliferation and EMT by targeting DUSP16 and activating the JNK/p38 MAPK signaling pathway. This study reveals a novel underlying mechanism of OS progression and provided a new strategy for treatment of OS. However, the pathogenesis of osteosarcoma still needs more research to elucidate.

## Supplementary Information

**Additional file 1:** **Table S1.** Expression of miR-769-5p and DUSP16 according to patients’ clinical features.

**Additional file 2: Table S2.** Primer sequences used in this research for qRT‐PCR.

**Additional file 3:** **Table S3.** Details of primary antibodies applied in this study.

**Additional file 4:** **Table S4.** Details of top 5 miRNAs in GSE28423.

**Additional file 5:** **Figure S1.** Research methodology flowchart.

## Data Availability

Most of the datasets supporting the conclusions of this article are included within this article and the additional files. The datasets used or analyzed during the current study are available on reasonable request.

## Abbreviations

OS: Osteosarcoma
BMSC: Bone mesenchymal stem cells
miRNA: MicroRNA
DUSP16: Dual-specific phosphatase 16
EMT: Epithelial-mesenchymal transition
qRT-PCR: Real-time quantitative polymerase chain reaction
WB: Western blot
IHC: Immunohistochemistry
HE: Hematoxylin–eosin
