# Supplementary material for: Exosomal transfer of miR-769-5p promotes osteosarcoma proliferation and metastasis by targeting DUSP16
Source: Cancer Cell Int. 2021 Oct 18;21:541. doi: 10.1186/s12935-021-02257-4 (PMC8522039; doi:10.1186/s12935-021-02257-4)
Supplement: Supplementary file 1 — Additional file 1: Table S1. Expression of miR-769-5p and DUSP16 according to patients’ clinical features. [file 12935_2021_2257_MOESM1_ESM.docx]

**Table S1** Expression of miR-769-5p and DUSP16 according to patients’ clinical features

|  |  | miR-769-5p expression | |  | DUSP16 expression | |  |
| --- | --- | --- | --- | --- | --- | --- | --- |
| Characteristics | Number | High group | Low group | P value | High group | Low group | P value |
| Age(y) |  |  |  |  |  |  |  |
| <18 | 30 | 17 | 13 | 0.594 | 13 | 17 | 0.443 |
| ≥18 | 34 | 17 | 17 |  | 18 | 16 |  |
| Gender |  |  |  |  |  |  |  |
| Female | 32 | 16 | 16 | 0.616 | 16 | 16 | 0.802 |
| Male | 32 | 18 | 14 |  | 15 | 17 |  |
| Location |  |  |  |  |  |  |  |
| Femur/Tibia | 47 | 26 | 21 | 0.559 | 22 | 25 | 0.665 |
| Elsewhere | 17 | 8 | 9 |  | 9 | 8 |  |
| TNM stage |  |  |  |  |  |  |  |
| I | 34 | 13 | 21 | 0.011^a^ | 21 | 13 | 0.023^a^ |
| II/III | 30 | 21 | 9 |  | 10 | 20 |  |
| Tumor size(cm) |  |  |  |  |  |  |  |
| <5 | 31 | 12 | 19 | 0.025^a^ | 20 | 11 | 0.013^a^ |
| ≥5 | 33 | 22 | 11 |  | 11 | 22 |  |
| Lung Metastasis |  |  |  |  |  |  |  |
| Yes | 28 | 20 | 8 | 0.01^a^ | 9 | 19 | 0.021^a^ |
| No | 36 | 14 | 22 |  | 22 | 14 |  |
| ^a^P<0.05(Chi-square test) |  |  |  |  |  |  |  |
