# Supplementary material for: Exosomal transfer of miR-769-5p promotes osteosarcoma proliferation and metastasis by targeting DUSP16
Source: Cancer Cell Int. 2021 Oct 18;21:541. doi: 10.1186/s12935-021-02257-4 (PMC8522039; doi:10.1186/s12935-021-02257-4)
Supplement: Supplementary file 2 — Additional file 2: Table S2. Primer sequences used in this research for qRT‐PCR. [file 12935_2021_2257_MOESM2_ESM.docx]

**Table S2** Primers of genes in this research for qRT‐PCR

|  | **Forward** | **Reserve** |
| --- | --- | --- |
| **DUSP16** | ACAGCAATAAGCTCAAGCGTTC | AGTCTGCTCCGATAGTTCCT |
| **U6** | CTCGCTTCGGCAGCACA | AACGCTTCACGAATTTGCGT |
| **β-actin** | GTGCACCTGACTCCTGAGGA | CTTGATACCAACCTGCCCAG |
| **hsa-miR-769-5p** | AACAAGTGAGACCTCTGGGT | GTCGTATCCAGTGCAGGGT |
| **hsa-miR-18a** | CCAAGGTAAGGTGCATCTAGTG | CAGTGCAGGGTCCGAGGTAT |
| **hsa-miR-9** | GCCCGCTCTTTGGTTATCTAG | CTCGCTTCGGCAGCACA |
| **hsa-miR-301a** | CCAGTGCAATAGTATTG | CTCAACTGGTGTCGTGGA |
| **hsa-miR-31** | ACGGCAAGATGCTGGGCA | CAGTGCTGGTCCGAGTGA |
