# Supplementary material for: Exosomal transfer of miR-769-5p promotes osteosarcoma proliferation and metastasis by targeting DUSP16
Source: Cancer Cell Int. 2021 Oct 18;21:541. doi: 10.1186/s12935-021-02257-4 (PMC8522039; doi:10.1186/s12935-021-02257-4)
Supplement: Supplementary file 3 — Additional file 3: Table S3. Details of primary antibodies applied in this study. [file 12935_2021_2257_MOESM3_ESM.docx]

**Table S3.** details of primary antibodies applied in this study

| **Gene specificity** | **Manufacture of primary antibody** | **Dilution rate**  **(WB)** | **Dilution rate**  **(IHC)** | **Specificity** | **Catalog number** |
| --- | --- | --- | --- | --- | --- |
| DUSP16 | Proteintech | 1：500 | 1：200 | Rabbit | 14237-1-AP |
| c-Myc | Proteintech | 1：1000 |  | Rabbit | 10828-1-AP |
| CDK4 | Proteintech | 1：1000 |  | Rabbit | 11026-1-AP |
| cyclin D1 | Proteintech | 1：1000 |  | Rabbit | 26939-1-AP |
| N-cadherin | CST | 1：1000 |  | Rabbit | 13116S |
| Vimentin | CST | 1：1000 |  | Rabbit | 5741S |
| E-cadherin | CST | 1：1000 |  | Rabbit | 3195S |
| phospho-ERK | CST | 1：1000 |  | Rabbit | 8544S |
| ERK | CST | 1：1000 |  | Rabbit | 4695T |
| phospho-p38 | CST | 1：1000 |  | Rabbit | 4511T |
| p38 | CST | 1：1000 |  | Rabbit | 8690T |
| phospho-JNK | CST | 1：1000 |  | Rabbit | 4668T |
| JNK | CST | 1：1000 |  | Rabbit | 9252T |
| CD9 | CST | 1：1000 |  | Rabbit | 13403S |
| CD81 | CST | 1：1000 |  | Rabbit | 52892S |
| calnexin | CST | 1：1000 |  | Rabbit | 2679T |
| Ki-67 | Abcam |  | 1：200 | Rabbit | ab16667 |
| CD63 | Abcam | 1：1000 |  | Rabbit | ab216130 |
| β-Actin | CST | 1：1000 |  | Rabbit | 4970T |
