# Supplementary material for: Exosomal transfer of miR-769-5p promotes osteosarcoma proliferation and metastasis by targeting DUSP16
Source: Cancer Cell Int. 2021 Oct 18;21:541. doi: 10.1186/s12935-021-02257-4 (PMC8522039; doi:10.1186/s12935-021-02257-4)
Supplement: Supplementary file 4 — Additional file 4: Table S4. Details of top 5 miRNAs in GSE28423. [file 12935_2021_2257_MOESM4_ESM.docx]

**Table S4** Top 5 miRNAs in GSE28423

| **id** | **logFC** | **P.Value** |
| --- | --- | --- |
| **hsa-miR-18a** | 6.644988 | 4.81E-10 |
| **hsa-miR-9** | 6.082743 | 6.52E-06 |
| **hsa-miR-769-5p** | 5.63262 | 9.49E-10 |
| **hsa-miR-301a** | 5.575997 | 4.05E-07 |
| **hsa-miR-31** | 5.474493 | 0.003444 |
