# Supplementary figures and images for: Exosomal transfer of miR-769-5p promotes osteosarcoma proliferation and metastasis by targeting DUSP16
Source: Cancer Cell Int. 2021 Oct 18;21:541. doi: 10.1186/s12935-021-02257-4 (PMC8522039; doi:10.1186/s12935-021-02257-4)

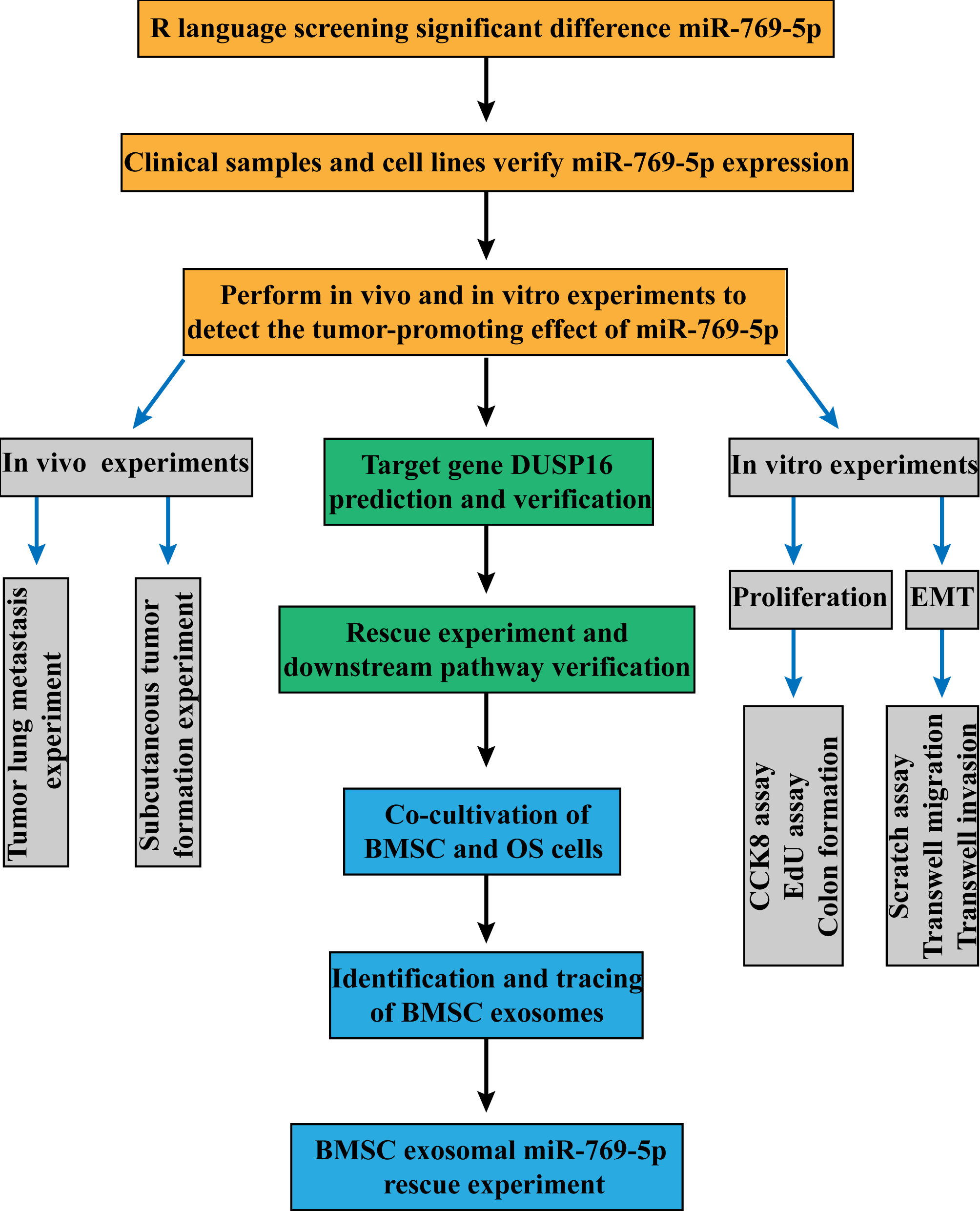

Supplement: Supplementary file 5 — Additional file 5: Figure S1. Research methodology flowchart. [file 12935_2021_2257_MOESM5_ESM.tif]
